# Hidden history of the tobacco BY-2 cell line

**DOI:** 10.1007/s10265-023-01490-4

**Published:** 2023-08-29

**Authors:** Toshiyuki Nagata

**Affiliations:** Faculty of Bioscience and Applied Chemistry, Hosei University, 3-7-2 Kajinocho, Koganei-shi, Tokyo, 189-5587 Japan

**Keywords:** *Agrobacterium tumefaciens*, Cell syncrony, Tobacco BY-2 cells, Transformation

## Abstract

For almost 50 years, tobacco (*Nicotiana tabacum*) BY-2 cells have been widely recognized as an important cell line for plant biology. The cell line grows rapidly, can be synchronized to a high degree, and is excellent for imaging; over the years, these features have led to many high-impact discoveries. However, certain other uses of this cell line are virtually unknown. In the early days, I was involved in distributing the cells to laboratories around the world. Many of these scientists wanted to study the cell cycle; however, I also distributed the cells to scientists who were elucidating the mechanism of plant transformation by *Agrobacterium tumefaciens*. In fact, BY-2 cells played an essential role in the identification and analysis of *Vir* genes on the Ti plasmid; likewise, the cells were important for discovering the factor that induces the expression of *Vir* genes. Thus, BY-2 cells were crucial for the development of modern plant biotechnology. Here, I recount the story of how this came to pass and explain why the use of BY-2 cells in this work was never recognized.

## Content

It seems to be widely accepted that the tobacco (*Nicotiana tabacum* L.) BY-2 cell line is a model cell line of vascular plants; however, there are a few stories concerning the line that have never been told. From time to time, this secrecy may cause confusion or misunderstanding. If I don’t tell these stories now, they are likely to remain unknown forever. Thus, I dare to tell them on this occasion.

## Encounter

In the first chapter of a monograph on tobacco BY-2 cells, I described how I encountered the tobacco BY-2 cell line (Nagata 2004). To avoid duplicating material from that article, here I will write some additional personal experiences.

In the last week of March 1981, I was asked by Prof. Yoshimi Okada, Department of Biochem. & Biophys., University of Tokyo, to demonstrate the delivery of TMV (Tobacco Mosaic Virus) RNA encapsulated in liposomes to protoplasts prepared from Madagascar periwinkle (*Catharanthus roseus*) cultured cells. This was conducted under a research program supported by Ministry of Education and Science of Japan. Dr. Seiichiro Hasezawa, then a PhD student of University of Tokyo, assisted me in this work, while Dr. Yuichiro Watanabe, then an undergraduate student, attended this course as a participant. In this experiment, delivery of viral RNA into protoplasts was assessed by staining with an FITC-labeled antibody against TMV after culture for 24 h (Fukunaga et al. 1981). To detect this staining, fluorescence microscopy was conducted in the lab of Prof. Tetsuo Iino, Department of Biological Sciences, University of Tokyo.

The experiment ran successfully; however, I was asked to repeat the experiment with a certain tobacco cell line, whose source was unknown to me at that time. RNA delivery to protoplasts from this line also succeeded; however, the level of infection was not as high as with the *C. roseus* cells and there was room for improvement. I procured a culture bottle of this cell line and brought it back to Nagoya University, where I had been working since May 1979. At that time, as my family was still living in Tokyo, I spent this weekend in Tokyo and came back with the cells to Nagoya by bullet train on the next Monday.

When I started to culture the cells by myself, I found an unusually high growth rate. Although the infection of BY-2 protoplasts was slightly lower than that of *C. roseus* protoplasts in the beginning, the introduction of a new cellulase Onozuka RS instead of Onozuka R10 brought a significant increase of infection. The cellulase Onozuka RS was found to have stronger activity in removing cell walls from BY-2 cells, resulting in the later commercialization of this enzyme by Yakult Corp. With this cell line, we successfully improved the protocol so that liposome-encapsulated TMV-RNA could be introduced readily into BY-2 protoplasts. I then asked a colleague of Prof. Okada, Dr. Koji Ohno (lecturer, and later appointed Prof. of Plant Mol. Biol., Hokkaido University, who passed away in 1993), what was the source of this tobacco cell line. He answered that it was called “tobacco BY-2” and was given to Prof. Okada by the Japan Tobacco & Salt Monopoly Public Corp. I next asked Dr. Takashi Matsumoto of the Research Institute of Japan Tobacco & Salt Monopoly Public Corporation for permission to use the cell line. However, his answer was surprising: No, because the Corporation had not offered the cell line to Okada’s lab. Evidently, the cell line had been transferred secretly. Nonetheless, finally I was allowed to use the cell line after rather tough negotiations, but I was not allowed to give the cell line to anybody else. Our results on RNA uptake were soon published (Nagata et al. 1981). Several publications of these experiments showed effective delivery of nucleic acids into protoplasts, papers that were widely noticed (Nagata 1987a, b).

In May 1982, I was invited to attend and give a lecture at the Miles International Symposium, held at Johns Hopkins University, Baltimore. This symposium was extremely interesting, because people from animal, plant, and microbial fields came together and discussed common issues of transformation and fusion of cells (Nagata 1984). I took advantage of the invitation to extend my work in this area; I spent 5 weeks in the USA collaborating with Profs. Milton Gordon and Eugene Nester of University of Washington, Seattle. Unusual for plant scientists, both belonged to the School of Medicine of University of Washington. Also, I stayed at Calgene (now subsidiary of Monsanto Co.) for a week, and a day at the International Plant Research Institute at Palo Alto, California. Although I was not allowed officially to give the BY-2 cells to others, I left the cell line at Seattle in the name of NT-1. This ambiguous name was selected because it could mean either *Nicotiana tabacum* or Nagata, Toshiyuki.

However, my attempt at clandestine distribution appears not to have been so successful, at least based on some anecdotal reports. The first was from a researcher of International Rice Res. Institute (IRRI), Manila, Dr. Osmat Azzam, who earned a PhD at University of Wisconsin, and who visited me at University of Tokyo in the mid 1990s. She asked me: What is the difference between the BY-2 line and NT-1 line. She explained that in her hands the propagation of Gemini virus (a DNA virus) in the two cell lines was very different: well propagated in BY-2, very slow in NT-1. My answer to her was that both should be the same. The other report was from scientists at Dow Chemical Co. around 2004. They use cell lines to produce certain types of animal antigen and again they found NT-1 performing much more poorly than BY-2. I suspect these observations reflect sub-optimal maintenance of NT-1, and a consequent slowdown of cell division. Propagation of DNA viruses depends on cell division as does general biosynthesis.

## Cell synchrony

After I found unusually high growth rate of tobacco BY-2 cells, the idea fell upon me to try to synchronize the cells with aphidicolin, which had been obtained from Dr. A. Todd of ICI UK shortly before. Doing so, I found very beautiful synchrony of the cells in the very first trial, results I eventually published (Nagata et al. 1982). Generously, over several years, Dr. Todd gave me a total of around half a gram of aphidicolin, for which I am very much obliged. Cell synchrony of BY-2 cells with aphidicolin was established by 1982 and this synchrony was far superior to that of other lines. For example, the synchrony of *C. roseus* was considered rather high; however, the peak mitotic index for synchronized *C. roseus* cells is around 10 to15% but the peak for BY-2 is 70 to 80% and in fact was later shown with a two-step procedure combining aphidicolin and propyzamide to reach 95%.

Around the time of my first publications with BY-2 cells (Nagata et al. 1981, 1982, 1987; Okada et al. 1986), Dr. Hiroh Shibaoka, Prof. of Osaka University (who unfortunately passed away in 2018 from a hiking accident) asked me how to synchronize BY-2 cells. His reasoning seemed to be as follows: Our method showed an extremely high cell synchrony ratio; however, many labs in Japan tried, but could not reproduce our level of synchrony. This generated a lot of whispering to the effect that Nagata’s method was fishy. Nonetheless, Dr Shibaoka hesitated to believe such whispers because he trusted me, going back to when we met while I was an undergraduate student at University of Tokyo. So, Dr. Shibaoka sent his staff to my lab to learn the synchrony method first-hand. I was at that time an Associate Professor at the National Institute of Basic Biology, Okazaki.

The first to come was Dr. Tatsuo Kakimoto (now professor at Osaka University), who visited me in the 1st week of January 1987. As he was very smart, he learned how to do the synchrony and he said: Yes! he could do it in Osaka. However, after 2–3 weeks, I heard from Osaka that they still couldn’t repeat the method. Subsequently, Dr. Seiji Sonobe (now at Hyogo Prefectural University) came to bring back the cells back to Osaka. This was repeated many times and some others visited me at Okazaki. Finally, after half a year they could reproduce our method. It turned out that the most important point was how to maintain the cells. The answer was so simple, as anybody can repeat my protocol, if they culture the cells properly! At the optimal condition, BY-2 cells can be maintained with an inoculum size of 2% introduced into fresh medium, once a week.

The next problem was to get permission for the Osaka group to use the cells. This became possible with the help of Dr. Nobutaka Takahashi (Prof. Emeritus of University of Tokyo, who passed away in 2016). The reason why the Japan Tobacco Corp. (by this time renamed from the Japan Tobacco & Salt Monopoly Public Corp.) wanted to restrict the use of BY-2 cells was related to a patent for mass propagation. At one time, they were growing the cells in a 15 ton tank at the Corporation’s Odawara Laboratory. They intended to blend cultured BY-2 cells with tobacco leaves for cigarette production. However, these blends persistently smelled bad, and they gave up this project reluctantly. Probably because of this fact, the Japan Tobacco Co. eventually allowed the cells to be distributed. Then the cells spread to many places in Japan. Shortly after that, Shibaoka’s group succeeded in preparing functional phragmoplasts from the synchronized protoplasts (Asada et al. 1991), which caused world-wide amazement.

Shortly after that, Profs. Anne-Marie Lambert and Claude Gigot, Institut de Biologie Moléculaire des Plantes, CNRS (Centre National de la Recherche Scientifique), Strasbourg contacted me to learn the synchrony of BY-2; and so I have been to Strasbourg, France, quite often and several times I needed to replenish their supply of cells. Their cell problems turned out to be caused by the fact that they used a kind of transfer pipette connected to an aspiration machine, which seemed to damage the transferred cells. To improve matters, several times, I brought them a graduated glass pipette, with a large mouth, attached to an autoclavable silicon bulb. With cells and synchrony method now established, Lambert and colleagues studied cytoskeleton dynamics during cell division, while Gigot and colleagues examined the oscillation of cell cycle genes. Somewhat later, Dr. Gigot passed away with a heart attack in 1998, while he was in Greece for his summer holidays. Now it became certain that BY-2 cells are a useful tool for examining the cell cycle dependence of certain genes, which had been mostly isolated from plant cells by homology with yeast cell cycle genes. This success became a standard to evaluate the cell cycle dependence of certain genes isolated from various kinds of plant materials.

Next, BY-2 cells went to Ghent University, Ghent, Belgium, where I have been more than 10 times. Further, they spread to Bordeaux University, Institut des Sciences Végétales, CNRS at Gif-sur-Yvette, France, University of Cambridge, UK, VTT in Helsinki, Finland and other places in Europe. Around that time, as I was asked to write a review paper for the *International Review of Cytology*, I contributed an article, in which among more conventional topics, I wrote up how to culture BY-2 cells properly (Nagata et al. 1992). Nonetheless, even after this publication, certain strange papers on the synchronization of BY-2 cells appeared in journals.

## Origin of BY-2 cells

The most frequent question about BY-2 cells that I receive is: What is their origin? The line was produced from the tobacco cultivar Bright Yellow 2. Bright Yellow cultivars (there are several) were introduced from the USA by Murai Corp. in 1906, before the tobacco industry became monopolized by the Japanese government. Subsequently, BY-1, -2, -3, and ‐ 4 were bred. In fact, BY-4 is still cultivated in Kumamoto Prefecture. Dr. Nobumaro Kawashima of the Japan Monopoly Corp. was the first to raise cultured cells from a BY-2 plant; however, he did not publish any paper. His main scientific contribution was the characterization of so-called “Fraction I protein” (now understood to be rubisco) in the lab of Prof. Samuel G. Wildman, University of California, whom I met in 1978 in Los Angeles one year before his retirement. 　The first publications on BY-2 cells appeared in 1972, with staff of Japan Tobacco & Salt Monopoly Public Corp. as authors, but said little about how they made the line (Kato et al. 1972a). In 1975, a paper where authors from the Japan Tobacco & Salt Monopoly Public Corporation were joined by Dr. Shiro Nagai, a microbiologist from University of Tokyo states that the line came from callus that had been induced from a seedling (Kato et al. 1972b, 1975). Apparently, seedlings were simply placed on callus-inductive agar medium and, after a few rounds of subculture, transferred to liquid culture. However, it is not recorded how many cultures were needed to find the unique BY-2 line or whether steps were taken to induce genetic instability. As people at the Japan Tobacco & Salt Monopoly Public Corp. were mostly biochemists and chemical engineers, they did not seem to recognize the importance of the specific steps used to induce the line.

Japan Tobacco & Salt Monopoly Public Corp. intended to propagate cells to produce material with reduced nicotine, which could be mixed with tobacco leaves for producing cigarettes. To begin, they constructed a 1500-liter culture vessel and then they built a 15 ton tank, in which they cultured cells for 66 days. Although mass production was plentiful, the products retained a strange, protein-like smell, which could not be removed by any means, and they finally abandoned this project. Around that time, some labs officially obtained the BY-2 line, while other labs obtained the line secretly. Regarding this, I was apparently the first person outside of the Corporation to recognize the unusual high growth rate of this BY-2 cell line and to establish its high synchrony, accomplishments of which I am proud.

To conclude this section, I would like to mention a cell line derived from BY-2 and named 2B-13. The 2B-13 cells grew without auxin for at least 30 years. This auxin autotrophy was established after BY-2 cells were irradiated with UV light, which should cause mutations, but the details of the irradiations were not specified. This cell line was also produced by the Japan Tobacco & Salt Monopoly Public Corp. so as to be able to culture the cells without the addition of 2,4-D. To examine the auxin autotrophic nature of 2B-13, we did some preliminary work (Shimizu et al. 2006). Then I wanted to characterize genetic difference between the two at the molecular level; however, around that time, I was fully occupied with administrative tasks for starting new Faculty of Bioscience at Hosei University, which was my new job after retirement from The University of Tokyo. During that time, 2B-13 was lost owing to a careless mistake by coworkers. Thus, I couldn’t find any clues to search for determining auxin autotrophy with this cell line. My lesson from this is that maintenance of cell lines is crucial and that I should not trust anybody for this matter.

## Secret role of BY-2 cells in plant biotechnology

In 1982, the same year that I attended the Miles International Conference in Baltimore (mentioned above), I also visited labs in Europe and South Korea. During that year, I met Dr. Gyn An (later Prof. of Biology, Pohang Univ., S. Korea) at four places around the globe: Seattle, Nottingham, Seoul, and Nagoya. He graduated from Seoul National University and then got a PhD in Canada, along with a Centenary grant offered from the Canadian Government that supported him in working anywhere in the world. Towards the end of 1982, he visited me at Nagoya University, staying for 2 months while we worked together. When he came back to the University of Washington, he brought back the BY-2 cells with him to Seattle. A few years later, Dr. An published that BY-2 cells are readily susceptible to transformation with *A. tumefaciens* (An 1985).

Right around the time that Dr. An brought BY-2 cells to Seattle, Dr. Scott Stachel moved there to join the crown gall group int Seattle. Although his former boss, Dr. Howard Goodman, moved to Massachusetts General Hospital in Boston, Dr. Stachel wouldn’t move to the East Coast. In Seattle, he constructed an interesting plasmid, comprising a Tn7 transposon linked to the lacZ operon. He reasoned that after introduction of this plasmid into the Ti plasmid of *Agrobacterium tumefaciens*, the *Vir* regions of the Ti plasmid could be determined based on random transposon jumps, and in this way when plant cells would be infected by the various transformed *Agrobacterium* lines, the function of *Vir* regions could be monitored by means of LacZ activity. However, he found no suitable plant materials for this trial until he tried BY-2 cells, which were amenable. Thus, the functions of *Vir* locus genes were discovered by means of this experimental system (Stachel et al. 1985a).

In summary, through this work, all *Vir* loci of the Ti plasmid were determined and mapped to regions outside of the T-DNA in the Ti plasmid. Then it was shown that upon expression of these loci, the T-DNA was cut out from the Ti plasmid and formed a complex with certain Vir proteins. Subsequently the complex was introduced into plant cells, resulting in the integration of T-DNA into the chromosomes of recipient plant cells (Nester et al. 1984; Stachel et al. 1985b).

For this cornerstone of plant biotechnology, in the seminal paper elucidating the *Vir* loci (Stachel and Nester 1986), the involvement of BY-2 cells was never stated directly. Nonetheless, if one carefully reads the paper, it is clear that BY-2 cells were indeed used.

Shortly after that, further events occurred where the starring role of BY-2 cells was again omitted from the credits. Now, the next question was what factor was responsible for the expressions of *Vir* genes. Just around that time, Dr. Stachel moved to the lab of Prof. Marc Van Montagu, Ghent University, Belgium. Soon thereafter, they published a report identifying the factor inducing *Vir* genes, purified from culture filtrates of *A. tumefaciens* and tobacco leaves, as acetosyringone, a report that appeared in *Nature* (Stachel et al. 1985b). According to information told to me by Prof. Milton Gordon (who passed away in 2005) but not recorded in the publication (Stachel and Nester 1986), Dr. Stachel found a clue to identify the factor from his work in Seattle with BY-2 cells; I have no direct evidence on this point.

Nevertheless, a few months later, the Seattle group published their paper, in *Science* (Bolton et al. 1986), which likewise identified acetosyringone as the crucial factor, but in this case the source material was a culture filtrate of *A. tumefaciens* and tobacco cells. Again, the name of BY-2 is missing. Thus, BY-2 cells were used to identify the functions of *Vir* genes and by at least one of the two studies discovering acetosyringone as a factor to induce *Vir* genes.

In view of the importance of these discoveries for plant biology, I gradually became motivated to tell these stories. This is my first time to do so. Many of the protagonists have passed away or are not particularly concerned about BY-2 cells. By the way, I heard that Dr. Stachel came back to the USA and joined the lab of Prof. Richard Axel, Columbia University, whom I met at the first meeting on recombinant DNA between Japan and USA in 1980 and at the Miles International Conference in 1982, mentioned above. Later Prof. Axel became a Nobel laureate.

Commenting further about these happenings, studies on the formation of crown galls on plants infected with *Agrobacterium tumefaciens* were a most intriguing subject (e.g. gene exchange between eukaryotic cells and prokaryotes, or horizontal gene transfer) and consequently an extremely competitive situation arose, for example, Seattle group vs. Ghent group vs. a 3rd group at Leiden University, in the Netherlands. There were three main issues; first was proof of the transformation of plant cells or the presence of T-DNA within the plant genome, which was done by Dr. Mary-Dell Chilton (University of Washington, Seattle, then Washington University, St. Louis, now at Roche Institute, S. Carolina). This news was brought to me in 1977 by a Christmas card from Dr. Milton Gordon. The second issue was the identification of *Vir* gene functions and the identification of the factor that induces *Vir* genes, in both of which tobacco BY-2 played an important role. The third issue was the use of this system for the delivery of foreign genes into plants; namely the start of gene manipulation in plants. For this third issue, a background story has been published recently (Heimann 2018), documenting the essential contributions of Profs. Marc Van Montagu and Jeff Schell. I add that I have also had close interactions with Jeff and Marc at many occasions. The story of BY-2 cells is not included in this book (Heimann 2018), of course.

Thus, for the development of plant biotechnology, BY-2 cells being extremely susceptible to *A. tumefaciens*, were used for several key experiments but left out by name from the Materials and Methods sections, an omission that has not been recognized previously. I think such happenings are very rare. Perhaps many people would ask: Why do I disclose these stories now? One answer may be the fact that in my current situation, there is no reason to keep these secret further, as I am now Prof. Emeritus at The University of Tokyo and also at Hosei University.

## And now

As described above, BY-2 cells have played important roles in various aspects in plant science; however, some of these stories have not been known publicly. This is a strong reason to write this essay. Thus, this is not a scientific paper, but a kind of personal memoir. Now it is not easy to follow the full details of BY-2 cells and new stories are emerging around the world. In this situation, I put some stress on the fact that inappropriate handling of the cells easily changes its original characteristics as mentioned in the case of NT-1 cells. This could be another issue to be studied further. If anybody would have interests to follow and know the fate of BY-2 cells, I would respond to these inquires. Recently, I leaned that Prof. Tobias Baskin of University of Massachusetts would like to follow the fate of BY-2 cells; I am keen to learn about his findings and I am thankful. Further, I would like to add that Prof. Baskin helped me edit this essay.
